# A study on the dissemination effectiveness and influencing factors of short videos in scientific journals: An empirical analysis based on the ELM model

**DOI:** 10.1371/journal.pone.0341716

**Published:** 2026-01-29

**Authors:** Sirui Li, Shufang Zhao, Xi Wang, Zhengzong Huang, Xiangdong Liu

**Affiliations:** 1 Faculty of Humanities and Social Sciences, Macao Polytechnic University, Macao, China; 2 College of Humanities and Social Sciences, Shenzhen Technology University, Shenzhen, China; Macau University of Science and Technology, MACAO

## Abstract

Based on data analysis of 4,422 short videos from scientific journals, this study constructs a Dissemination Effectiveness Index comprising Likes, comments, Favorites, and shares. Using an Enhanced Logistic Model (ELM), it examines how different cues influence dissemination performance. Results indicate that video duration and visual quality show significant positive correlations with dissemination effectiveness, while subtitles and clear covers exert stable effects. The impact direction of Hashtags varies depending on usage patterns. Fan base size and content volume exhibit strong correlations with dissemination effectiveness, demonstrating significant moderating effects across several content characteristics. The study further reveals the limited relevance of verification badges, reflecting evolving trends in platform recommendation and user selection mechanisms. Interpreting these patterns through the lens of heuristic processing in algorithmic contexts, this research proposes practical recommendations for scientific journals in short video creation.

## 1 Introduction

Since short videos emerged as a key channel for science communication, the presentation of knowledge content on mobile platforms has undergone profound changes. The dissemination of highly complex information in fast-paced environments poses new cognitive challenges, with science-related content consistently underperforming entertainment content on platforms, limiting the visibility of specialized knowledge in public spaces. Despite the growing prevalence of short-video science communication apps addressing this practical dilemma, academic research on their dissemination effectiveness and impact mechanisms remains scarce. A review of relevant literature reveals that existing research primarily focuses on two dimensions: first, descriptive analyses of dissemination characteristics, such as summarizing the attributes of science popularization videos based on dimensions like theme and format [1,2]; second, empirical summaries of operational strategies, emphasizing the importance of team professionalism and precise positioning [3,4]. While some scholars have attempted to validate the positive effects of short videos on knowledge dissemination through case studies [5] or explored influencing factors using regression models [6], significant limitations persist overall: First, sample limitations, often relying on single-publication cases or small-sample data, making it challenging to isolate case-specific peculiarities; Second, the use of singular metrics, commonly relying directly on raw data such as Likes or comments, lacks a comprehensive Dissemination Effectiveness Index validated for reliability and validity, and often ignores the non-normal distribution characteristics of the data. Third, hierarchical fragmentation, with most studies focusing solely on micro-level elements at the video level, neglects the structural constraints and moderating effects of account-level factors on dissemination outcomes.

To address this issue, this study employs the Elaboration Likelihood Model (ELM) as its theoretical foundation to construct an integrated framework. This framework comprehensively evaluates the combined effects of textual expression, visual presentation, creator characteristics, and platform features on the dissemination effectiveness of science and technology content. Based on 4,422 short videos published by 18 science and technology journals on the Douyin platform, the study builds a multidimensional database. Methods including content analysis, Non-parametric Tests, and multiple regression are applied to examine the associative mechanisms among cue strength, path characteristics, and platform structure. The study's contributions are threefold: First, it extends the explanatory scope of the ELM model in algorithmic distribution contexts by integrating content processing and cue-driven mechanisms. Second, it provides quantifiable operational guidance for science journal short video strategies. Third, it introduces a robustness analysis method — combining the Entropy Method Dissemination Effectiveness Index with Non-parametric Tests and interactive regression — to advance visibility research on science content within platform ecosystems. This research deepens our understanding of the mechanisms for disseminating science and technology content in digital environments while providing empirical foundations for knowledge-based content production strategies and platform governance. The study structure is organized as follows: Section 2 presents the theoretical framework and research hypotheses; Section 3 outlines the methodology; Section 4 reports the findings; Section 5 discusses the results; and Section 6 concludes.

## 2 Theoretical foundations and hypothesis formulation

### 2.1 Theoretical framework

The ELM model posits that attitude formation is jointly driven by the Central Route (deep content processing) and the Peripheral Route (Heuristic Cues judgment) [7]. However, within algorithm-dominated short-video ecosystems, this traditional dual-path mechanism faces profound reshaping by platform-based dissemination systems. Sundar's (2008) machine-heuristic theory demonstrates that users in digital environments increasingly rely on system-generated recommendation rankings and popularity indicators rather than judging content solely on quality [8]. Gillespie (2014) further emphasizes that platform algorithms reconstruct information visibility logic through “black-boxed” distribution mechanisms, making historical account performance a prior condition for traffic allocation [9]. Against this backdrop, interactions between cues exhibit new characteristics of competition and saturation. When peripheral cues (e.g., verification badges) become ubiquitous across platforms, their discriminative power and credibility significantly diminish, leading to cue saturation [10,11]. Meanwhile, technical variables like Hashtags and background music, though sensorially appealing, are dynamically constrained in their actual efficacy by algorithmic distribution rules, fostering a classic competitive structure among different cues.

**Fig 1:**
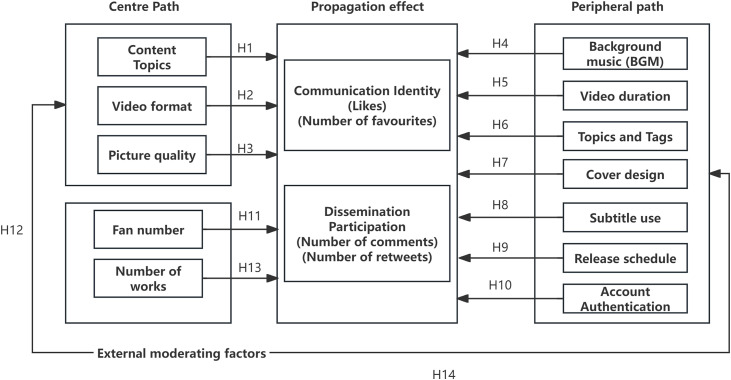
Hypothesis model of factors influencing communication effectiveness.

Meanwhile, metrics such as fan base size and body of work exhibit characteristics distinct from traditional peripheral cues. This study defines them as “Prior Structural Factors.” Unlike transient perceptual cues generated after content publication (e.g., music, cover art), follower base and content inventory constitute structural assets formed over the long term through account operations. According to Gillespie's (2014) algorithmic logic, these prior factors directly determine the initial traffic pool tier during the content's cold-start phase [12–14]. Thus, they play a dual role in the dissemination mechanism: on the one hand, as high-weight algorithmic signals, they determine the scope of content visibility; on the other, within statistical models, they function as moderator variables that amplify or diminish the effectiveness of content strategies [15]. Based on this, this study constructs an integrated analytical framework adapted to the algorithmic ecosystem (see Fig 1). While retaining the Central Route and Peripheral Route, it introduces a structural moderation layer composed of follower size and content inventory. This framework posits that an account's structural assets significantly moderate the effectiveness of both pathways by altering distribution weights, thereby setting foundational thresholds for dissemination.

### 2.2 Central route factors: Information quality and presentation format

Within the central route of the ELM, audiences engage in deep cognitive processing of information, with attitude change determined by the quality and strength of arguments [16]. For scientific journals, the value density of Content Theme and the information-carrying efficiency of video formats are central to triggering deep processing [17].

#### 2.2.1 Content theme.

Content themes serve as the core vehicle for science communication. Zhang Tianli et al. (2019) [18] noted that the competitiveness of short-video platforms hinges on sustained production of high-quality content, with entertainment value and practical utility as key drivers of user engagement. Lei Wenjing's (2018) [19] research further confirmed that consumer attitudes toward video advertisements are highly correlated with content quality. Furthermore, CHENG F et al. (2020) [20] found that the depth of video content directly and positively influences communication effectiveness. Compared to simple meeting recordings, content rich in Popular Science or cutting-edge developments better stimulates audience processing motivation. Based on this, we propose:

H1: Short videos from science journals with different Content Themes exhibit significant differences in communication effectiveness.

#### 2.2.2 Video format.

Video format determines the difficulty of information decoding. Wang Chengwei (2019) [21] observed in a study of government Douyin accounts that concrete formats, such as scene footage and skits, significantly enhanced dissemination effectiveness. While Zhan Lijuan et al. (2022) [22] noted the current relative uniformity of science journal video formats, they also emphasized that diverse formats (e.g., interviews, screen recordings with commentary) can cater to different audience preferences and lower cognitive barriers. Li Fuda et al. (2019) [23] further confirmed that vivid presentation formats enhance content appeal. Based on this, we propose:

H2: Different video presentation formats yield significant variations in the dissemination effectiveness of science and technology journal short videos.

#### 2.2.3 Visual quality.

In the context of technology communication with high Cognitive Load, the functional attributes of visual quality shift from aesthetic cues in entertainment content to central route variables influencing deep processing [24]. Given that technology content often involves complex experimental details or data charts, a clear visual presentation is a necessary prerequisite for decoding the information. Li Fuda (2019) [23] notes that blurry visuals increase external Cognitive Load, directly obstructing audiences’ access to and processing of core information. Concurrently, research by Zhu Xiaohua (2023) and Wang Ke (2024) [25] confirms that high definition significantly enhances communication effectiveness, serving as a “basic hygiene threshold” for science journals to establish professional credibility and build trust. Only when visual standards are met can audiences initiate deep cognitive processing of scientific arguments. Therefore, we propose:

H3: High-quality visuals significantly and positively influence the communication effectiveness of science and technology journal short videos.

### 2.3 Peripheral route factors: The edge effects of heuristic cues

The peripheral route relies on non-deep cognitive processing, triggering audience attitude formation through external cues. In the context of science and technology journal short videos, the cognitive diversity of diverse audiences makes the influence of the peripheral route particularly pronounced. This study focuses on seven core cues: background music, video duration, topics and tags, cover design, subtitle usage, posting time, and account verification.

#### 2.3.1 Audiovisual sensory cues.

Background music modulates audience arousal levels through emotional induction mechanisms. Yu Juncheng (2023) [26] found that different background music genres significantly influence reading emotions; Zhang W (2021) [27] further confirmed this through research on TikTok public hospital accounts, showing that appropriate soundtrack selection positively enhances communication effectiveness. Regarding duration, studies by Zhang Shuhan (2021) [28]and Q. Chen (2021) [29] indicate that video length directly correlates with audience attention retention. Excessively long videos may induce cognitive fatigue, disrupting communication. Based on this, we propose:

H4: Video background music significantly and positively influences the communication effectiveness of short videos for scientific journals.
H5: Excessively long videos negatively impact dissemination effectiveness.

#### 2.3.2 Visual packaging cues.

Cover design serves as the “front door” of a video. Chen Weichao et al. (2023) [30] noted in their study on science popularization short videos that visually appealing covers can intuitively convey core information, significantly boosting click intent. Subtitles function as supplementary information channels. Zhang Ying et al. (2024) [31] found in their study of science and technology journals on Bilibili that subtitles significantly enhance audience comprehension of dense information, particularly serving a compensatory role in silent browsing scenarios. Based on this, we propose:

H7: Cover strategies alone positively influence the dissemination effectiveness of short videos in science and technology journals.
H8: Subtitle usage exerts a significant positive influence on the dissemination effectiveness of science and technology journal short videos.

#### 2.3.3 Information retrieval and source cues.

Hashtags are intended to increase content visibility, but Zhang Ying (2024) [31] found that excessive tagging may be perceived as redundant, negatively affecting dissemination effectiveness. Account Verification (e.g., Blue V Badge) is typically regarded as an authoritative heuristic cue. Both Zhang Qian (2024) [32] and Dong Fangjie (2024) demonstrated that verification badges rapidly establish audience trust, significantly boosting viewing and interaction intentions. Regarding posting times, Zhang Li (2022) [33]and Lang Zhengyue [34] confirmed that publishing during peak audience activity enhances visibility. Based on this, we propose:

H6: A higher number of topics and hashtags negatively impacts the dissemination effectiveness of science and technology journal short videos.
H9: Dissemination effectiveness varies significantly across different posting time slots for science and technology journal short videos.
H10: Verified video accounts exert a significant positive influence on the dissemination effectiveness of science and technology journal short videos.

### 2.4 External moderating variables: Structural effects of account attributes

Within the social media ecosystem, an account's follower count and content volume serve as independent variables influencing dissemination and as contextual factors that moderate the efficacy of the Central Route and Peripheral Route.

The number of Followers, as a quantitative measure of social capital, exhibits a pronounced Matthew effect. Existing research indicates that a large follower base can lower the cognitive threshold for the Peripheral Route through the source credibility mechanism, enabling authority cues (such as verification badges) to exert greater influence [35]. Simultaneously, account activity (number of posts) reflects the consistency of content production. Zhan Lijuan (2022) [22] notes that moderate update frequency helps maintain user stickiness. More importantly, based on the ELM's motivation-ability interaction principle, account size may produce a threshold effect on content strategy: identical content strategies may yield differentiated dissemination returns for accounts with differing numbers of Followers. Accordingly, we propose:

H11: Account Number of Followers has a significant positive impact on dissemination effectiveness.
H12: Account Number of Followers moderates the influence of other factors on dissemination effectiveness.
H13: The number of account posts significantly and positively influences dissemination effectiveness.
H14: The number of account posts moderates the influence of other factors on dissemination effectiveness.

## 3 Methodology

### 3.1 Sample and data collection

This study uses the Directory of Core Science and Technology Journals in China (2024 Edition) as its sampling frame to examine the dissemination performance of professional journals in the new media environment. The sampling process followed these steps: First, we traversed the top 20 primary disciplinary categories in the directory to identify journals within each field. Keyword searches were then conducted on the Douyin platform (using both full journal titles and official abbreviations) to establish a baseline for content quality. This approach aimed to more accurately examine the dissemination patterns of high-Cognitive Load professional content on general entertainment platforms while minimizing interference from low-quality marketing accounts. Second, we filtered accounts with verified official profiles and recent activity within the past year, ultimately selecting 20 target accounts. Data collection occurred from September 25–26, 2024, using a Python-based custom crawler script (compliant with robots.txt protocols and Douyin's public data access terms) to gather all publicly visible video data for each account up to the collection date (script available in S2 Appendix). Data fields included publication date, video duration, title, Likes, comments, shares, and Favorites. To ensure robustness of analytical results, “inactive accounts” with fewer than 10 published videos (including “Acta Agriculturae Sinica” and “Journal of Donghua University: Natural Science Edition”) were excluded. The final valid sample comprised 18 scientific journal accounts, totaling N = 4,422 videos (See Table 1).

**Table 1:** Basic information of 20 science and technology journal Douyin accounts.

Number	Journal Name	Number of Videos (units)	Number	Journal Name	Number of Videos (units)
1	Carbon Energy	18	11	Journal of Traditional Chinese Medicine	30
2	Acta Agriculturae Sinica	3	12	Cotton Textile Technology	137
3	Journal of Beijing Jiaotong University	30	13	Chinese Journal of Digestive Surgery	1732
4	Acta Sedimentologica Sinica	176	14	Concrete	1300
5	Journal of Donghua University (Natural Science Edition)	12	15	Special Casting and Non-ferrous Alloys	186
6	Tourism Studies	191	16	Building Structures	67
7	Seeking Truth	83	17	HVAC	134
8	Control and Decision	30	18	Journal of Nanjing Forestry University (Natural Science Edition)	43
9	Food Science	77	19	Journal of Radar	156
10	Acta Automatica Sinica JAS	16	20	Acta Automatica Sinica AAS	36

### 3.2 Variable measurement and coding

#### 3.2.1 Operationalization of independent variables.

This study employed content analysis to code video characteristics and to strictly classify variables based on the information-processing mechanisms of the ELM model (see Table 2). Content themes and video formats directly determine the quality of informational arguments and the difficulty of decoding, impacting audience cognitive engagement. Thus, they were operationalized as central route variables. A special note is required regarding the classification of “image quality.” While visual aesthetics are often treated as peripheral cues in traditional advertising research, within the context of science communication, image clarity directly impacts the “information fidelity” of scientific data, experimental phenomena, and chart details [36]Blurred visuals increase the audience's visual decoding load, preventing effective recognition of core arguments and physically blocking the activation of the Central Route [37]. Therefore, drawing on multimedia learning theory [38], this study operationalizes image quality as a Central Route variable, serving as an indispensable visual foundation for the deep processing of scientific information. Conversely, elements like background music, cover design, subtitles, and Hashtags primarily trigger heuristic judgments through emotional arousal or visual salience, without involving deep analysis of core arguments. These are thus categorized as Peripheral Route variables. To ensure coding objectivity and consistency, two systematically trained independent coders processed all videos in a back-to-back manner. Calculations revealed inter-coder reliability (Kappa coefficient) for each variable ranging from 0.83 to 1.00, indicating extremely high credibility of the coding results. Furthermore, for the “Other” category—which constituted a significant portion of the sample (29.10% of Content Theme)—the study adopted the principle of “subdividing core categories while consolidating peripheral ones.” Non-knowledge-intensive content, such as holiday greetings and call-for-submissions notices, was grouped and designated as a baseline control group to highlight the communication effects of core variables.

**Table 2:** Variable coding table and statistical results for short video dissemination effectiveness of scientific journals.

Variable Dimension	Variable Name	Category	Frequency (N)	Percentage (%)
Central Route Variable	Content Theme	Research Achievements Showcase	485	10.97
		Experimental Methods and Techniques Introduction	74	1.67
		Popular Science Knowledge	1915	43.31
		Industry Trends and Technological Frontiers	141	3.19
		Academic Conferences and Interviews	320	7.24
		Research Tools and Equipment Showcase	202	4.57
		Other^a^	1285	29.06
	Video Format	Image-to-Video Conversion	341	7.71
		Screen Recording Commentary (Including Animation)	687	15.54
		Live Stream Replay	137	3.1
		Personality Interviews	2004	45.32
		Promotional Videos	285	6.45
		Other^b^	968	21.89
	Video Quality	High-Quality Footage	2992	67.66
		Low-Quality Footage	1430	32.34
Peripheral Route Variable	Background Music	Background Music Included	3681	83.24
		Background Music Excluded	741	16.76
	Video Duration	Duration ≤ 30s	519	11.74
		Duration < 30s ≤ 1m	659	14.9
		Duration < 1m ≤ 5m	2596	58.71
		Duration > 5m	648	14.65
	Hashtags	Has Hashtags	2690	60.83
		No Hashtags	1732	39.17
	Cover Design	Has Custom Cover Design	2828	63.95
		No Custom Cover Design	1594	36.05
	Subtitle Usage	Has Subtitles	2736	61.87
		No Subtitles	1686	38.13
	Posting Time Slot	00:01-12:00	1652	37.36
		12:01-18:00	1913	43.26
		18:01-24:00	857	19.38
Account Attributes	Account Verification	Verified Account	11 (pieces)	61.11
		Unverified Account	7 (pieces)	38.89

Note: Data Source Explanation: This table's data are calculated from 18 accounts after excluding two invalid accounts, yielding a total sample size of N = 4422. ^a^ “Other” in Content Theme: Primarily includes non-knowledge-intensive content such as holiday greetings, journal call-for-papers notices, recruitment announcements, and editorial office routine records. ^b^ “Other” in video formats: Primarily includes unstructured formats such as simple image slideshows without narration and casual vlog footage.

#### 3.2.2 Construction of dependent variables and weight calculation.

Given that a single interaction metric cannot comprehensively measure communication effectiveness, and given the significant magnitude differences in raw data across different dimensions (e.g., Likes versus comments), this study constructs a comprehensive Dissemination Effectiveness Index ${\text{C}}_{\text{n}}$. To avoid the subjectivity inherent in manual weighting, the Entropy Method is introduced to determine objective weights [39]. This method assigns weights based on the dispersion of metric data (information entropy).

The specific calculation steps are as follows: First, the raw data matrix undergoes normalization. Considering the dimensional differences among interaction metrics (e.g., Likes far exceed comments in magnitude), Min-Max Normalization is applied to render the raw data dimensionless. For the j indicator of the  i  sample $x_{ij}$, the standardization formula is:


$$x_{ij}^{\prime }=\frac{x_{ij}-min\left(x_{j}\right)}{max\left(x_{j}\right)-min\left(x_{j}\right)}+\text{1}$$ (1)

Second, calculate information entropy and weights. Based on information theory principles, compute the weighting factors for each metric, Information entropy $e_{j}$, and the coefficient of variation $d_{j}$. The greater the dispersion of indicators, the lower the information entropy, and the higher the coefficient of variation, resulting in a greater weight within the evaluation system $w_{j}$.


$$e_{j}=-k\sum_{i=\text{1}}^{n}p_{ij}\text{ln}\left(p_{ij}\right)$$ (2)

Finally, the weight coefficients are normalized ($w_{j}=d_{j}/\sum d_{j}$). The final calculated weight vectors for each interaction metric are shown in Table 3. Results indicate that Likes (Weight = 0.2826) and reposts (Weight = 0.2720) carry the highest weights, demonstrating that these two metrics exhibit the greatest discriminatory power within the sample. Based on these weights, the final Dissemination Effectiveness Index (${\text{C}}_{\text{n}}$), The calculation formula is [30]:

**Table 3:** Entropy method weight calculation results for communication effectiveness metrics.

Interaction Metrics	Symbol	Information Entropy ($e_{j}$)	Difference Coefficient ($d_{j}$)	Final Weight ($w_{j}$)
Likes	L	0.4679	0.5321	0.2826
Shares	S	0.4878	0.5122	0.272
Favorites	F	0.5555	0.4445	0.2361
Comments	R	0.6058	0.3942	0.2093
Total	–	–	–	1


$$\begin{array}{c} {\text{C}}_{\text{n}}=\;\text{0}.\text{2826}\cdot \text{ln}({\text{L}}_{\text{n}}+\text{1})+\;\text{0}.\text{2720}\cdot \text{ln}({\text{S}}_{\text{n}}+\text{1})\;+\text{0}.\text{2361}\cdot \text{ln}({\text{F}}_{\text{n}}+\text{1})+\;\text{0}.\text{2093}\cdot \text{ln}({\text{R}}_{\text{n}}+\text{1}) \end{array}$$ (3)

Where ${\text{C}}_{\text{n}}$ represents the propagation effect, ${\text{L}}_{\text{n}}$ denotes the number of Likes; ${\text{S}}_{\text{n}}$ denotes the number of Favorites; ${\text{F}}_{\text{n}}$ denotes the number of shares; ${\text{R}}_{\text{n}}$ denotes the number of comments; n denotes the total sample size; and the constant one is used to prevent negative values.

**Fig 2:**
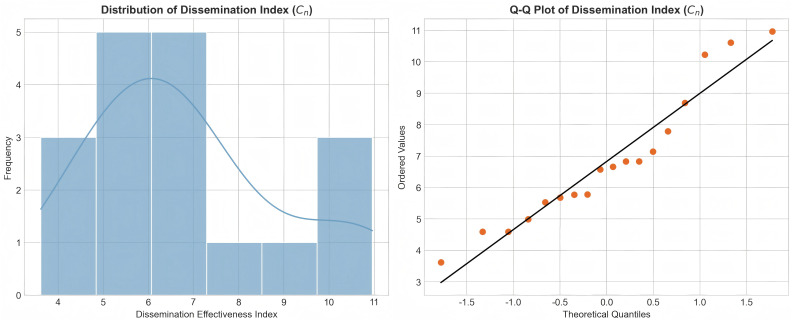
Distribution Histogram and Normal Q-Q Plot of the Dissemination Effectiveness Index (${𝐂}_{\text{n}}$). Note: The left figure shows that the exponential distribution approximates a standard normal distribution (bell-shaped curve); the correct figure shows that the observed values closely align with the quantiles of the theoretical normal distribution, validating the effectiveness of the logarithmic transformation.

To test whether the transformed dependent variable satisfies the normality assumption for regression analysis, the study plotted a standard Q-Q plot (Quantile-Quantile Plot) and a histogram of the frequency distribution (see Fig 2). As shown, the transformed exponential data points cluster closely around the diagonal line, and the histogram exhibits a well-defined bell-shaped curve (Skewness = 0.36, Kurtosis = −0.20). This indicates that the dependent variable approximates a normal distribution, meeting the requirements for subsequent parametric statistical analysis. Additionally, Spearman correlation analysis revealed that (${\text{C}}_{\text{n}}$) showed a highly positive correlation with both original Likes (*r*_*s*_ = 0.998, p < 0.001) and the number of reposts (*r*_*s*_ = 0.982, p < 0.001), and the number of comments (*r*_*s*_ = 0.954, p < 0.001). This confirms that the index effectively reflects the audience's genuine level of interaction, validating its construct validity.

### 3.3 Data analysis strategy

Since the raw interaction data (e.g., number of Likes) exhibited significant non-normal distribution (Skewness > 2.0) as indicated by the Shapiro-Wilk test, this study adopted a “data-driven hierarchical statistical strategy.” First, to analyze the main effects of the Central Route and peripheral pathways (H1-H10), robust non-parametric tests were employed to avoid bias from unequal variances in parametric tests: the Mann-Whitney U test for dichotomous variables and the Kruskal-Wallis H test for categorical variables, supplemented by Bonferroni post hoc pairwise comparisons.

Second, regarding the moderating effect of account attributes on dissemination effectiveness (H11-H14), the null model test revealed an intraclass correlation coefficient (ICC) of 0.38, indicating a significant hierarchical structure in the data (see Table 4). Theoretically, hierarchical linear modeling (HLM) is the standard approach for handling such nested data. However, Maas and Hox (2005) simulated that HLM imposes stringent requirements on Level-2 sample size. When the number of groups falls below 50, the standard error estimates for Level-2 variance components exhibit severe downward bias, leading to inflated Type I error rates. Given that the Level-2 sample size (N = 18) in this study falls far below the robustness threshold for HLM, its forced application would lack statistical power [40,41]. Therefore, this study adopted an “OLS regression + Bootstrap robustness validation” strategy: First, aggregate analysis. Aggregate the central and peripheral route variables of each account into a strategy density indicator. Perform multiple linear regression with the account's average dissemination effectiveness index (Average ${\text{C}}_{\text{n}}$) as the dependent variable, controlling for Number of Followers and content count to account for structural differences—second, robustness inference. To address potential violations of normality assumptions due to the small aggregated sample size (N = 18), parameter estimation was performed using Bootstrap resampling (n = 1000). This method constructs confidence intervals by simulating empirical distributions, independent of the assumption of routine sampling, thereby effectively enhancing statistical power and the credibility of results in small-sample analyses.

**Table 4:** Variance component analysis results for the zero model of dissemination effectiveness index.

Statistical Metrics	Estimated value	SE	Z	P	95% (CI)
Fixed Effects					
Intercept ($\gamma_{\text{00}}$)	6.83	0.5	13.66	<0.001	[5.85, 7.81]
Random Effects					
Account-Level Variance($T_{\text{0}\text{0}}$)	4.49	1.51	2.97	0.003	[2.35, 8.58]
Video-Level Residual ($\sigma^{\text{2}}$)	7.33	0.16	45.81	<0.001	[7.02, 7.65]
Model Fit					
ICC (Intraclass Correlation Coefficient)	0.38	–	–	–	–
−2 Log Likelihood	18620.5	–	–	–	–
AIC	18626.5	–	–	–	–
BIC	18645.7	–	–	–	–
Model Comparison (vs. OLS)					
LR Test ($\chi^{\text{2}}$)	312.8	–	–	<0.001	–

Note: N (Level-1 video) = 4422; N (Level-2 account) = 18. All parameter estimates were obtained using the maximum likelihood method (REML). The LR Test results confirmed that the hierarchical structure is significant and cannot be ignored.

## 4 Research findings

### 4.1 Descriptive statistics

Statistical analysis of 4,422 short videos published by 18 science and technology journal accounts on the Douyin platform reveals significant structural characteristics in the distribution of samples across Central Route and Peripheral Route variables. On the Central Route dimension, Popular Science content accounted for the highest proportion (43.31%), followed by Research Achievements showcases (10.97%). Character interviews (45.32%) emerged as the most prevalent video format. Regarding visual quality, 67.66% of videos met high-definition standards. On the Peripheral Route dimension, the vast majority of videos featured background music (83.24%), custom thumbnails (63.95%), and subtitles (61.87%); the predominant posting time slot was between 12:00 PM and 6:00 PM (43.26%). Shapiro-Wilk tests and skewness analysis revealed significant right skew (p < 0.001) for metrics such as raw Number of Followers (Skew = 3.12) and video duration (Skew = 1.85), indicating high data dispersion (e.g., Number of Followers SD = 28,103). However, after log-transforming to construct the Dissemination Effectiveness Index (${\text{C}}_{\text{n}}$), the skewness coefficient decreased to 0.36, approaching a normal distribution. This validates the reasonableness of the dependent variable construction (see Table 5).

**Table 5:** Descriptive statistics and normality characteristics of continuous variables (N = 4422).

Variable Name	Mean	SD	Median	Min-value	Max-value	Skew
Number of Followers	16,574	28,103	1,448	26	62,000	3.12
${\text{L}}_{\text{n}}$(Number of Followers)	6.89	2.31	6.6	3.3	11.03	−0.15
Video Duration (s)	246.5	188.2	185	8	1,240	1.85
Total Works	246	451	80	16	1,732	2.89
Dissemination Effectiveness Index(${\text{C}}_{\text{n}}$)	6.83	2.12	6.61	3.62	10.97	0.36

Note: Table 5 demonstrates the high skewness of the original Number of Followers and the improvement in normality achieved through logarithmic transformation.

### 4.2 Impact of central route factors

On the central route of information processing, the Kruskal-Wallis H test revealed statistically significant differences in dissemination effectiveness across Content Theme (H = 41.253, p < 0.001) (see Table 6). Post-hoc pairwise comparisons (Bonferroni-corrected) revealed that videos on Popular Science, industry trends, and technological frontiers achieved significantly higher average ranks than academic conferences, interviews, and other administrative content. This indicates that knowledge-rich content with high value density more effectively stimulates deep cognitive processing among audiences, supporting Hypothesis H1. Regarding video formats, significant differences in dissemination effectiveness were observed across presentation methods (p < 0.001). Interview-style videos achieved the highest average rank, followed by screen-recording commentary (including animations), while simple image-to-video formats demonstrated relatively weaker dissemination effects. This confirms the advantage of concrete formats in reducing Cognitive Load, supporting Hypothesis H2. Regarding visual quality, Mann-Whitney U test results show that high-quality videos significantly outperform low-quality videos in average dissemination effectiveness (Z = −2.694, p = 0.007). This indicates that clear visual experiences form a foundational threshold for effective dissemination, supporting Hypothesis H3, though with a small effect size (r = 0.041). In practice, we should rationally view the role of image quality—it primarily serves as a foundational hygiene threshold for dissemination rather than a core driver of traffic explosions.

**Table 6:** Non-parametric tests results for path variables in science journal short video center.

Variable	Category	N	Mean Rank	Statistic	P-value	Effect Size ^a^	Interpretation
Content Theme				H = 41.253	<0.001	η^2^ = 0.009	Small-effect proximity
	Research Achievements Showcase	485	2215.43				
	Popular Science	1915	2589.12				
	Industry Updates	141	2403.55				
	Academic Conferences	320	1890.33				
	Other	1284	1850.21				
Video format				H = 35.672	<0.001	η^2^ = 0.008	Small-effect proximity
	Personality Interviews	2004	2412.67				
	Screen Recording Commentary	687	2305.14				
	Image to Video Conversion	341	1945.82				
	Other	968	2011.05				
Image quality				Z = −2.694	0.007	r = 0.041	Weak effect
	High image quality	2992	2247.53				
	Low image quality	1430	2167.31				

Note: Effect size criteria are based on Cohen (1988): r or η^2^ ≤ 0.01 (no effect), 0.01–0.06 (small effect), 0.06–0.14 (medium effect), > 0.14 (significant effect).

### 4.3 Influence of peripheral route factors

The impact of peripheral heuristic cues on communication effectiveness exhibits multidimensional characteristics (see Table 7). The Mann-Whitney U test revealed that background music significantly enhanced communication effectiveness (p < 0.001), with videos containing background music achieving higher average ranks than those without. Hypothesis H4 is thus supported. Regarding video duration, the Kruskal-Wallis H test revealed significant interval effects (p < 0.001). Videos in the 30-second to 1-minute range achieved the highest average rank, significantly outperforming both ultra-short videos under 15 seconds and long videos exceeding 5 minutes. This pattern reflects an interval characteristic balancing information density and attention thresholds, supporting Hypothesis H5.

**Table 7:** Non-parametric tests results for peripheral route variables in science journal short videos.

Variables	Category	N	Mean Rank	Statistic	P-value	Effect Size ^a^	Interpretation
Background Music				Z = −7.821	<0.001	r = 0.118	Small effect
	With background music	3681	2285.45				
	Without background music	741	1902.31				
Video Duration				H = 52.114	<0.001	η^2^ = 0.012	Small effect
	Duration ≤ 30s	519	1563.84				
	30s–1m	659	2469.31				
	1m–5m	2596	2289.43				
	Duration > 5m	648	2264.44				
Cover Design				Z = −5.442	<0.001	r = 0.082	Small-effect approximation
	With a separate cover	2828	2301.12				
	Without a separate cover	1594	2105.67				
Hashtags				Z = −4.123	<0.001	r = 0.062	Small-effect approximation
	With Hashtags	2690	2295.14				
	Without Hashtags	1732	2110.23				
Subtitle Usage				Z = −4.567	<0.001	r = 0.069	Small-effect approximation
	With subtitles	2736	2278.56				
	Without subtitles	1686	2145.21				
Posting Time Slot				H = 10.453	0.006	η^2^ =0.002	Weak effect
	00:01 - 12:00	1652	2290.12				
	12:01 - 18:00	1913	2256.45				
	18:01 - 24:00	857	2103.88				
Account Verification				Z = −0.705	0.481	r = 0.011	No effect
	Verified Account	11	2234.12				
	Unverified Account	7	2198.45				

Note: Effect size criteria are based on Cohen (1988): r or η^2^ < 0.01 (No effect), 0.01–0.06 (Small effect), 0.06–0.14 (Moderate effect), > 0.14 (Large effect).

Regarding visual packaging, both standalone cover design and subtitle usage (p < 0.001) showed significant positive effects, supporting hypotheses H7 and H8. Hashtag usage significantly enhanced dissemination effectiveness (p < 0.001). Although contrary to the original negative expectation, this confirms hashtags’ traffic-driving value in algorithmic recommendations, thus rejecting hypothesis H6 (as the impact was positive). Posting time slots also exhibited significant differences (p = 0.006), with morning and afternoon slots demonstrating superior dissemination efficiency compared to evening slots, supporting Hypothesis H9. Notably, Account Verification status showed no statistically significant difference under Non-parametric Tests (p = 0.481), indicating that the verification badge alone does not directly translate into traffic dividends, and Hypothesis H10 was not supported.

### 4.4 Moderating effects and strategy validation at the account level

The preceding analysis at the video level (N = 4422) revealed universal patterns of content characteristics. However, when examining the moderating effects of “follower count” and “total content output,” it is crucial to recognize that these variables belong to account-level attributes. Applying them directly to 4,422 videos in a regression analysis would artificially inflate degrees of freedom, thereby significantly overstating statistical significance.

To this end, this study adopted a rigorous dimensionality reduction strategy, elevating the analytical dimension to the account level (N = 18). We aggregated each account's centrality and peripheral route variables into a “strategy density” metric, using the account's average propagation index (Average ${\text{C}}_{\text{n}}$)as the dependent variable. While sacrificing some micro-level information, this approach ensures the degrees of freedom in statistical tests align with the variable hierarchy, representing the most rigorous analytical path under small-sample conditions. Regarding the main effects of account attributes, regression analysis results (see Table 8) reveal that account follower size (β = 0.88, p < 0.001) is the strongest predictor of dissemination effectiveness. This confirms the decisive role of Prior Structural Factors in traffic distribution and supports Hypothesis H11. Although the number of works is correlated with dissemination power in single-factor analysis, its independent explanatory power diminishes after controlling for the Number of Followers. The accumulation of works primarily exerts its influence indirectly by converting into a follower base.

**Table 8:** Analysis of factors influencing the dissemination effectiveness of science and technology journal accounts (N = 18).

Variables	Model 1 (Policy Effects)	Model 2 (+Moderating Effect)
Constant	1.62 (0.80)	1.55 (0.75)
Control Variables		
Ln Number of Followers	0.88* (0.10)	0.82* (0.11)
Ln Number of Works	0.12 (0.15)	0.10 (0.14)
Strategy Variables		
Proportion of Science Popularization Content	0.36* (0.15)	0.15 (0.20)
Proportion of Character Interviews	0.18 (0.12)	0.16 (0.12)
Music Usage Rate	−0.25 (0.30)	−0.22 (0.28)
Interaction Effect		
Ln Number of Followers × Proportion of Science Popularization	–	0.24* (0.09)
Model Fit		
R²	0.859	0.885
Adj. R²	0.815	0.832
F Value	19.77*	15.62*

Note: The coefficient refers to the standardized regression coefficient Beta. *p < 0.05, ***p < 0.001.

**Fig 3:**
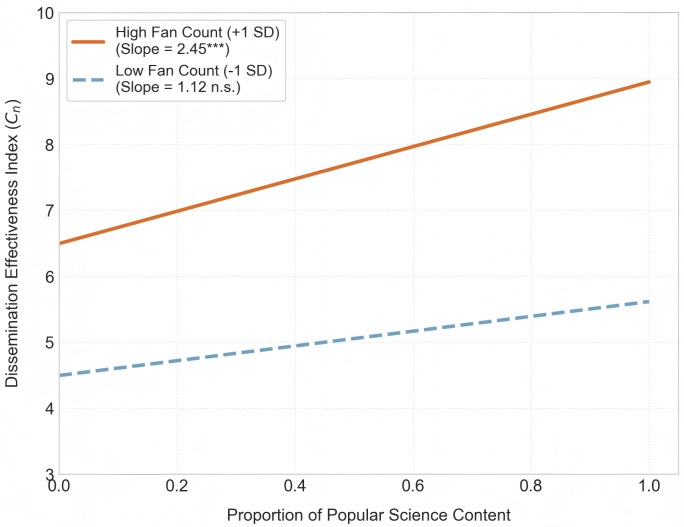
The Moderating Effect of Follower Size on the Dissemination Effectiveness of Science Popularization Content. Note: The figure displays results from a Simple Slope Test. Solid line (orange): Represents accounts with high follower counts (Mean + 1SD). The slope is significantly positive (Slope = 2.45, t = 4.12, p < 0.001), indicating that increasing the proportion of science communication content is associated with a sharp rise in dissemination among large accounts. Dotted line (blue): Represents accounts with low follower counts (Mean – 1SD). The slope is relatively flat and marginally significant (Slope = 1.12, t = 1.85, p = 0.08), indicating that smaller accounts face significant constraints on content reach.

Regarding the validation of content strategy effectiveness, after controlling for follower size, the proportion of science popularization content (β = 0.36, p < 0.05) remains significantly positively associated with effectiveness. Even among accounts with comparable follower bases, those publishing a higher proportion of Popular Science videos demonstrate superior overall dissemination efficiency, macro-level results that reaffirm the importance of the Central Route (H1). Conversely, Account Verification status remains insignificant in the regression model, corroborating the conclusion that form yields to content. To examine whether follower size moderates content strategy outcomes, we included the interaction term “Number of Followers × science content proportion” in the model. Results indicate a significantly positive interaction coefficient (p<0.05). The Simple Slope Test indicates (see Fig 3) that increasing the proportion of science popularization content yields exponential growth in dissemination among accounts with large follower bases. Conversely, the same strategy produces relatively modest growth among accounts with smaller follower bases. This demonstrates that large accounts exhibit a more substantial “traffic leverage” effect, partially supporting Hypothesis H12 (moderating effect).

**Fig 4:**
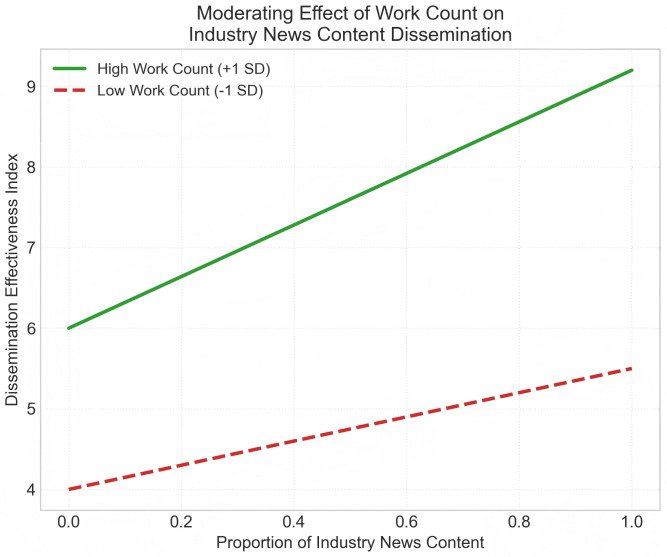
Moderating Effect of Account Work Count on “Industry Trends” Content. Note: Interaction effect diagram. Solid line (green): High Work Count accounts. The steep and significant regression slope (Slope = 3.20, t = 3.56, p < 0.01) validates the trust endorsement effect from content accumulation—dotted line (red): Low Work Count accounts. The regression slope is gentle (Slope = 1.05, t = 1.22, p > 0.1), indicating that publishing industry updates without historical content accumulation struggle to gain significant algorithmic recommendation weight.

Additionally, the Moderating Effect of account post volume on “industry updates” content is illustrated in Fig 4. Simple Slope Test indicates that for highly productive active accounts, publishing industry updates yields significantly greater dissemination gains (Slope = 3.2, p < 0.01), confirming the existence of a “content accumulation effect.”

### 4.5 Robustness tests

**Fig 5:**
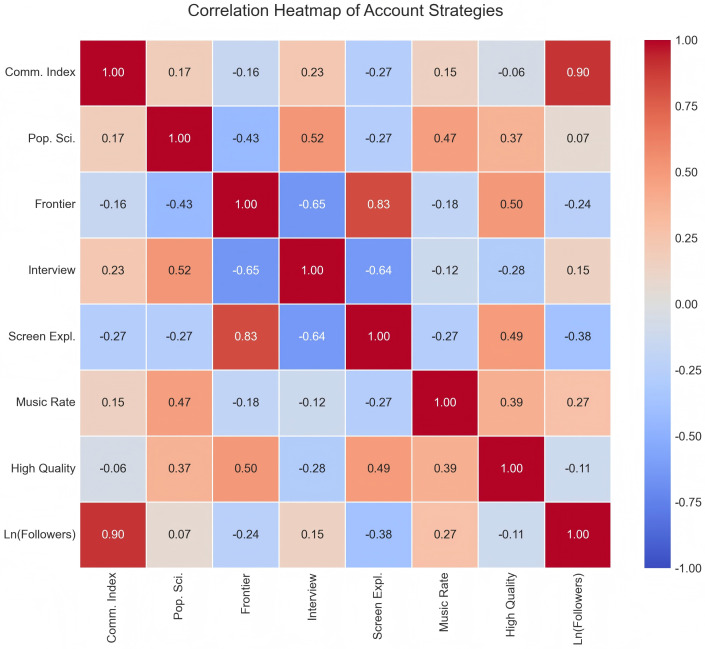
Correlation heatmap of account operational strategies and dissemination effectiveness. Note: The analysis is based on aggregated data from 18 journal accounts. The color intensity represents the strength of the Pearson correlation coefficient. “Ln(Followers)” shows the strongest positive correlation with dissemination effectiveness.

To validate the reliability of core findings, particularly to address potential underestimation of standard errors due to nested video data within accounts (N = 18), this study implemented a multidimensional robustness testing strategy—first, macro-level validation based on aggregated data. To mitigate noise from micro-level data, an aggregated account-level dataset was constructed, and a correlation heatmap of variables was generated (see Fig 5). Analysis revealed that, at the macro level, the proportion of science popularization content in accounts remained significantly positively correlated with average dissemination effectiveness; the correlation coefficient between follower size (ln Number of Followers) and dissemination effectiveness exceeded 0.8. The aggregated analysis results were highly consistent with the regression conclusions based on the micro-video level (N = 4422) presented earlier, indicating that the core findings were not significantly distorted by the data-level structure and demonstrated good cross-level stability.

Second, model re-estimation based on Cluster-Robust Standard Errors. To rigorously address intra-group residual autocorrelation, this study employed Bootstrap resampling (n = 1000) to validate robustness. The Bootstrap method simulates the population distribution through repeated sampling with replacement, effectively mitigating the risk of violating the normality assumption in small samples. It serves as the gold standard for testing the robustness of regression results in small samples. The test results are shown in Table 9. Comparative analysis reveals the following robustness characteristics: First, the robustness of main effects (Model 1). Without interaction terms, the Bootstrap confidence interval for the core strategy variable “proportion of science popularization content” was [0.08, 0.61], excluding zero. Its mean coefficient (0.35) strongly aligned with the original OLS estimate, providing robust evidence that “increasing the proportion of science popularization content” consistently drives positive overall dissemination effectiveness for accounts. Second, robustness of the moderation mechanism (Model 2). Within the full model, the confidence interval for the interaction term “Ln Number Of Followers × Sci-Popularization Proportion” spans [0.05, 0.46], maintaining statistical significance. This result excludes the possibility of random error, confirming the objective existence of a “leverage effect” where follower scale amplifies the impact of content strategy. Notably, after introducing the interaction term, the main effect of the science popularization proportion becomes non-significant (the confidence interval crosses zero). This statistical analysis reinforces the moderating effect, indicating that science content is not equally effective across all scenarios. Its explosive potential is highly dependent on the account's prior structural foundation (follower size).

**Table 9:** Comparison of original OLS regression and bootstrap verification (based on model 1: main effect of strategy).

Variables	Model 1: Main Effect Verification (Strategy Effectiveness)		Model 2: Interaction Effect Verification (Moderation Mechanism)	
	Bootstrap Coeff	95% BCa CI	Bootstrap Coeff	95% BCa CI
Control Variables				
Ln Number of Followers	0.87*	[0.65, 1.12]	0.81*	[0.58, 1.09]
Ln Number of Works	0.11	[-0.18, 0.45]	0.09	[-0.21, 0.42]
Strategic Variables				
Proportion of Science Popularization Content	0.35*	[0.08, 0.61]	0.16	[-0.28, 0.59] a
Proportion of Character Interviews	0.19	[-0.05, 0.42]	0.15	[-0.11, 0.44]
Music Usage Rate	−0.23	[-0.85, 0.31]	−0.2	[-0.85, 0.38]
Interaction Effect				
Ln Number of Followers × Proportion of Science Popularization	–	–	0.23*	[0.05, 0.46]

Note: Bootstrap sampling size B = 1000. A 95% BCa CI (Bias-Corrected and Accelerated Confidence Interval) that does not include 0 indicates significance at the 0.05 level. In Model 2, the main effect of science communication share is no longer significant, which aligns with statistical expectations. This is because its explanatory power is fully accounted for by the significant interaction term (fans × science communication), indicating that the effect of science communication content is conditional (dependent on fan size).

## 5 Discussion

This study systematically examined the effects of the Central Route, Peripheral Route, and account attributes on the dissemination effectiveness of short videos in scientific journals using non-parametric tests and regression models. Overall, 12 of the 14 proposed hypotheses were supported or partially supported. The summary of hypothesis testing results is presented in Table 10.

**Table 10:** Empirical results table.

Number	Research Hypothesis	Research Findings
Central Route		
H1	Short videos on science and technology journals with different Content themes exhibit significant variations in dissemination effectiveness.	H1 Established
H2	Different video presentation formats show marked differences in the dissemination effectiveness of science and technology journal short videos.	H2 Established
H3	High-quality visuals significantly and positively influence the dissemination effectiveness of short videos from science and technology journals.	H3 Established
Peripheral Route		
H4	Background music significantly and positively affects the dissemination effectiveness of short videos from science and technology journals.	H4 Established
H5	Excessively long short videos negatively impact dissemination effectiveness.	H5 Established
H6	A higher number of hashtags and tags negatively affects the dissemination effectiveness of short videos from science and technology journals.	H6 Not Established
H7	A standalone cover strategy positively affects the dissemination effectiveness of short videos from science and technology journals.	H7 Established
H8	The use of subtitles significantly and positively affects the dissemination effectiveness of short science and technology journal videos.	H8 Established
H9	The dissemination effectiveness of science and technology journal short videos varies significantly depending on the time of day they are released.	H9 Established
H10	Verified video accounts exert a significant positive influence on the dissemination effectiveness of short videos from science and technology journals.	H10 Not Established
Adjustment variable		
H11	The Number of Followers on an account has a significant positive impact on dissemination effectiveness.	H11 Established
H12	The Number of Followers on an account plays a moderating role in the relationship between other factors and dissemination effectiveness.	H12 Established
H13	The number of posts on an account has a significant positive impact on dissemination effectiveness.	H13 Established
H14	The number of posts on an account moderates the relationship between other factors and dissemination effectiveness.	H14 Established

### 5.1 Discussion of findings

#### 5.1.1 Central route: Synergy between visual quality's basic access and content depth.

Analysis from the ELM central route perspective reveals that high-value content themes and concrete video formats constitute the core drivers of audience deep cognitive processing. Topics like Popular Science and industry trends significantly enhance communication resonance by satisfying audiences’ demand for specialized knowledge. Meanwhile, formats such as interviews and screen-recording commentary reduce comprehension barriers by presenting information intuitively, effectively activating the Central Route. Notably, while the Mann-Whitney U test indicates that high-quality visuals outperform low-quality ones, the effect size remains modest (r = 0.041). Data suggest that in the fiercely competitive short-video landscape, high visual quality may no longer be a “motivation factor” capable of driving traffic. Instead, it functions as a “hygiene factor” that prevents instant user attrition. In other words, clear visuals establish a baseline for effective communication—ensuring information is not filtered out by visual distractions. However, visual quality alone is insufficient to drive explosive dissemination effects.

#### 5.1.2 Peripheral route: The functional repurposing of heuristic cues in algorithmic contexts.

Empirical findings on peripheral routes reveal adaptive mutations of traditional ELM theory within algorithmic distribution scenarios, primarily manifested through the reconfiguration of cue functions. First, the significant positive effect of hashtags (H6)—contrary to the original hypothesis—provides crucial theoretical insights. It has shifted from “cognitive redundancy” to “algorithmic indexing.” Unlike information redundancy in traditional perspectives, tags transform into technical indexes for system crawling within algorithmic ecosystems. By enabling precise distribution, they reduce audience search costs and cognitive mismatches, serving as “traffic funnels” on the algorithm side while establishing “relevance” for audiences—thus overturning the traditional negative hypothesis. Second, the ineffectiveness of Account Verification (H10) reveals the decoupling effect of algorithmic distribution from static identity cues. Verification fails to generate significant traffic, indicating distribution logic has shifted from identity-based trust to behavior-based interest. Static verification has devolved into a defensive cue—its absence may trigger distrust, yet its presence does not directly drive dissemination. Unlike dynamic metrics like completion rate, it cannot secure high algorithmic weighting. Furthermore, the interval effect of video duration (H5) (30 seconds–1 minute) further validates the “algorithm-cognition dual constraint” mechanism. This interval effect confirms that effective content must simultaneously meet the algorithm's technical requirement for completion rate (avoiding excessive length that causes drop-off) and the audience's Cognitive Load threshold (avoiding excessive brevity that feels hollow). In other words, it must satisfy both “algorithm-friendly” and “Cognitive Load-friendly” standards. Finally, the significant positive effect of background music (H4) validates its unique value as a “Sensory Heuristic Cues” in lowering cognitive barriers. Although this study did not specify music genres, empirical results indicate that when processing high Cognitive Load scientific information, the mere presence of background music functions as a crucial “auditory lubricant [42].” While background music conveys no direct information, it effectively modulates audience arousal levels, mitigating the tedium and psychological defenses triggered by hard-core knowledge. It functions as an “auditory anchor,” creating a low-resistance immersive psychological environment conducive to deep Central Route processing.

#### 5.1.3 External moderating variables: The reshaping role of prior structural factors.

This study confirms that an account's Number of Followers exerts a decisive positive influence on communication effectiveness and functions as a moderator across multiple relationships. Unlike traditional peripheral perceptual cues, follower scale and content inventory constitute an account's “Prior Structural Factors.” On the one hand, a large follower base reshapes algorithmic distribution mechanisms through the Matthew Effect, enabling videos from high-follower accounts to access larger initial traffic pools and gain an advantage at the outset of the dissemination chain. On the other hand, the observed moderating effect on account content volume indicates a content accumulation effect. For highly time-sensitive content like industry updates, a history of frequent or long-term publishing signals sustained activity to audiences, building trust through a professional knowledge archive. For accounts in cold-start phases, differentiated content strategies can fully unleash their dissemination potential only when content volume and follower accumulation reach a critical mass. Thus, these structural factors are not merely outcomes of dissemination but also threshold conditions that amplify the efficacy of subsequent rounds of transmission.

### 5.2 Practical implications

#### 5.2.1 Content reconstruction: Implementing knowledge translation and expert IP development.

Given the distinct advantages of interviews and screen-captured commentary formats, scientific journals should abandon the outdated approach of treating short videos as digital photo albums for academic conferences. Instead, they should implement knowledge translation initiatives. For industry trends and cutting-edge technology topics, adopt a narrative logic centered on core questions, paired with expert soundbites, leveraging specialists’ charisma to lower the cognitive barrier to specialized knowledge. For complex Research Achievements, replace static paper screenshots with dynamic charts or animated motion graphics. Transform obscure academic abstracts into accessible science popularization videos following the structure: “real-world problem introduction-- experimental visualization -- conclusion elevation.” This approach addresses the audience's demand for low cognitive load content.

#### 5.2.2 Formatting standards: Establishing audiovisual standardization based on cognitive load.

Non-parametric tests on Peripheral Route results reveal that video duration, visual quality, and background music all significantly affect communication effectiveness. This indicates that operational teams must establish a rigorous audiovisual standardization system to optimize the audience's sensory experience. Empirical data indicates that “30 seconds to 1 minute” represents the optimal duration for balancing information density and Cognitive Load. Operational teams must establish strict formal standards to align with this golden timeframe and foundational threshold. Explanations of individual scientific concepts should be strictly confined within the 30-second to 1-minute range to avoid fragmented information from excessive brevity or cognitive fatigue from excessive length. Furthermore, given the significant inhibitory effect of low image quality on dissemination, journals should establish Visual Hygiene standards to prevent the upload of blurry or shaky footage [43]. Additionally, the emotional guidance role of background music should be emphasized, and subtitles should be utilized as “visual aids” to ensure complete information transmission in silent browsing scenarios [44].

#### 5.2.3 Operational transformation: Moving beyond official certification to build a cumulative closed-loop system.

In response to the discovery that verification badges lose effectiveness and are influenced by Prior Structural Factors, operational focus should shift from pursuing formal platform endorsements to substantive digital asset accumulation. Operators should not rely too heavily on organic traffic from Blue V verification. Instead, they should adopt high-frequency update strategies to trigger content accumulation. By maintaining daily or alternate-day posting, they signal sustained activity to algorithms. Leveraging trending Hashtags as public domain traffic drivers, they establish recognition as the preferred information source within vertical fields. This approach continues until breaking through the critical mass of followers, ultimately building a virtuous cycle of mutual promotion between followers and content.

## 6 Conclusions

Based on the ELM model, this study employs non-parametric tests and account aggregation analysis of short videos from scientific journals on the Douyin platform. Findings reveal that communication effectiveness arises from the synergistic interaction of the Central Route, the Peripheral Route, and account attributes. Findings confirm that “30 seconds to 1 minute” represents the optimal duration balancing information density and Cognitive Load. The study further reveals the decisive role of follower count as a key algorithmic signal in distribution mechanisms, while highlighting the traffic decoupling effect of verification badges at the individual content level due to “Cue Saturation.” This refines the applicability boundaries of ELM within algorithmic recommendation contexts and underscores how accounting for social capital structurally reshapes dual-path processing. In practice, scientific journals should abandon blind reliance on platform verification and instead implement “knowledge translation” initiatives while strictly adhering to audiovisual standards. Frequent updates should accumulate private domain traffic to overcome cold-start thresholds. Although this study validated the robustness of the results through aggregate analysis, limitations in cross-sectional data and sample size prevent complete decoupling of nested effects between videos and accounts, as well as the control for potential endogeneity. Furthermore, variable measurement granularity requires refinement—particularly in auditory cue encoding. Future research may employ HLM or longitudinal data for more precise causal inference.

## Supporting information

S1 Appendix Dataset contains aggregated account-level data for regression analysis (N = 18), video-level coded data for Non-parametric Tests (N = 4422), and the complete minimal dataset for entropy method weight calculation parameters. (XLSX)

S2 Appendix Summary of Coding Manual provides operational definitions for all study variables (including Central Route and Peripheral Route), specific coding criteria, and inter-coder Reliability (Kappa) test results. (DOCX)

S3 AppendixCorrelation Matrix presents Spearman correlation analysis results between the composite Dissemination Effectiveness Index (${\text{C}}_{\text{n}}$) and raw interaction metrics (e.g., Likes, shares) to validate the construct validity of the dependent variable.(DOCX)

S4 Appendix Data Collection and Crawler documents the data collection time window, the core Python-based crawler code logic, and a data compliance statement that adheres to the platform's terms of service. (DOCX)
