# Supplementary material for: A study on the dissemination effectiveness and influencing factors of short videos in scientific journals: An empirical analysis based on the ELM model
Source: PLoS One. 2026 Jan 29;21(1):e0341716. doi: 10.1371/journal.pone.0341716 (PMC12854476; doi:10.1371/journal.pone.0341716)
Supplement: S2 Appendix — (DOCX) [file pone.0341716.s002.docx]

**S2 Appendix: Summary of Coding Manual**

To ensure the objectivity of content analysis, this study established detailed operational definitions for coding. Two coders conducted a pre-test on 100 samples before formal coding. Discrepancies (such as the distinction between "Popular Science" and "Research Achievements") underwent multiple rounds of discussion and revision. Ultimately, the inter-coder reliability (Cohen's Kappa) for all variables exceeded 0.83.

**Table A1 Operational Definitions and Coding Standards for Core Variables**

| Dimension | Variable Name | Code | Operational Criteria | Kappa |
| --- | --- | --- | --- | --- |
| Central Route | Content Theme | 1=Popular Science Knowledge | Explains scientific principles, natural phenomena, and everyday knowledge in an accessible language for the general public. | 0.92 |
|  |  | 2=Research Achievements | Presents specific research publications, experimental data, and patent breakthroughs with a strong academic focus. |  |
|  |  | 3=Industry Trends | Covers cutting-edge academic news, industry policies, and technology updates with emphasis on timeliness. |  |
|  |  | 4=Academic Conferences | Includes conference recordings, expert addresses, and lecture replays. |  |
|  |  | 5=Other | Holiday greetings, call for papers, editorial office routines, and simple scenic footage (reference group). |  |
|  | Video format | 1=Personality Interviews | The primary subject is a live person (expert/editor) speaking directly to the camera or being interviewed (Talking Head). | 0.95 |
|  |  | 2=Screen Recording Commentary | Uses PowerPoint presentations, animated demonstrations, or experiment screen recordings with voice-over narration but no live person on camera. |  |
|  |  | 3=Image-to-Video Conversion | Simple static image slideshow (PPT slide mode) lacking dynamic visual flow. |  |
|  |  | 4=Other | Pure text scrolls, Vlog-style casual footage, and other unstructured formats. |  |
|  | Image quality | 1=High Quality | Resolution ≥ 1080P, well-lit, sharp focus, stable footage without shaking. | 0.88 |
|  |  | 0=Low Quality | Resolution < 720P, blurry footage, dim lighting, or direct screen captures with moiré patterns. |  |
| Peripheral Route | Video Duration | 1= ≤ 30s | Ultra-short videos. | 1 |
|  |  | 2= 30s - 1m | Optimal duration range. |  |
|  |  | 3= 1m - 5m | Medium-length videos. |  |
|  |  | 4= => 5m | Long-form videos. |  |
|  | Background Music | 1=Yes | Full or partial background music (BGM) throughout the video. | 0.98 |
|  |  | 0=No | Only human voices or ambient sounds, no musical accompaniment. |  |
|  | Visual Packaging | 1=Cover image available | Cover art independently designed with title text and layout. | 0.94 |
|  |  | 0=No cover image | The system automatically captures a frame from the video as the cover, with no text. |  |
|  | Subtitle Usage | 1=Subtitles available | Video includes synchronized subtitles (including system-generated and proofread subtitles). | 0.96 |
|  |  | 0=No subtitles | No subtitles throughout, or only minimal keyword prompts. |  |
|  | Hashtags | 1=Available | Title or description includes hashtags preceded by the # symbol (e.g., #science #research). | 1 |
|  |  | 0=Not available | No hashtags used. |  |
