# Supplementary material for: A study on the dissemination effectiveness and influencing factors of short videos in scientific journals: An empirical analysis based on the ELM model
Source: PLoS One. 2026 Jan 29;21(1):e0341716. doi: 10.1371/journal.pone.0341716 (PMC12854476; doi:10.1371/journal.pone.0341716)
Supplement: S3 Appendix — (DOCX) [file pone.0341716.s003.docx]

**S3 Appendix: Correlation Matrix**

To verify the construct validity of the Dissemination Effectiveness Index ($\mathbf{C}_{\mathbf{n}}$), this study calculated Spearman's rank correlation coefficients between the DEE and each original interaction metric.

Spearman Correlation Matrix between DEE and Original Metrics (N=4422)

| Variables | 1. Likes | 2. Favorites | 3. Shares | 4. Comments | 5. Virality Index ($\mathbf{C}_{\mathbf{n}}$) |
| --- | --- | --- | --- | --- | --- |
| 1. Likes | 1 |  |  |  |  |
| 2. Favorites | 0.892*** | 1 |  |  |  |
| 3. Shares | 0.765*** | 0.814*** | 1 |  |  |
| 4. Comments | 0.841*** | 0.789*** | 0.723*** | 1 |  |
| 5. Virality Index ($\mathbf{C}_{\mathbf{n}}$) | 0.998* | 0.945* | 0.982* | 0.954* | 1 |

Note: ** p < 0.001 (two-tailed test). Results indicate a highly significant positive correlation with all original indicators, demonstrating its ability to effectively represent overall communication effectiveness.
