# Supplementary material for: A study on the dissemination effectiveness and influencing factors of short videos in scientific journals: An empirical analysis based on the ELM model
Source: PLoS One. 2026 Jan 29;21(1):e0341716. doi: 10.1371/journal.pone.0341716 (PMC12854476; doi:10.1371/journal.pone.0341716)
Supplement: S4 Appendix — (DOCX) [file pone.0341716.s004.docx]

**S4 Appendix: Data Collection & Crawler**

**1. Collection Time and Scope**

Collection Period: September 25, 2024 — September 26, 2024

Collection Targets: Official Douyin accounts of 18 target scientific journals.

Collection Content: All publicly visible historical video data (as of collection date).

**2. Crawler Tools and Logic**

This study employed Python 3.9 scripts utilizing `Selenium` to simulate browser behavior and `mitmproxy` packet capture technology for data acquisition. All operations strictly adhered to `robots.txt` protocols and platform-published data access rules, with no user privacy information collected.

**Core Code Snippet:**

import time

from selenium import webdriver

import pandas as pd

def scrape_journal_videos(account_url):

# Initialize browser driver

options = webdriver.ChromeOptions()

options.add_argument('--headless')

driver = webdriver.Chrome(options=options)

try:

driver.get(account_url)

video_list = []

last_height = driver.execute_script("return document.body.scrollHeight")

# Simulate scrolling to load all historical videos (Lazy Loading)

while True:

driver.execute_script("window.scrollTo(0, document.body.scrollHeight);")

time.sleep(2)

new_height = driver.execute_script("return document.body.scrollHeight")

if new_height == last_height:

break

last_height = new_height

elements = driver.find_elements_by_css_selector(".video-card")

for el in elements:

video_data = {

"title": el.find_element_by_class("title").text,

"likes": parse_number(el.find_element_by_class("like-count").text),

"duration": el.find_element_by_class("duration").text,

"publish_time": el.find_element_by_class("time").text

}

video_list.append(video_data)

finally:

driver.quit()

return pd.DataFrame(video_list)

# Data Collection Time：September 25, 2024 — September 26, 2024
